# MicroRNA-98 Inhibits Hepatic Stellate Cell Activation and Attenuates Liver Fibrosis by Regulating HLF Expression

**DOI:** 10.3389/fcell.2020.00513

**Published:** 2020-06-19

**Authors:** Qi Wang, Song Wei, Haoming Zhou, Lei Li, Shun Zhou, Chengyu Shi, Yong Shi, Jiannan Qiu, Ling Lu

**Affiliations:** ^1^School of Medicine, Southeast University, Nanjing, China; ^2^Hepatobiliary Center, The First Affiliated Hospital of Nanjing Medical University, Nanjing, China; ^3^Research Unit of Liver Transplantation and Transplant Immunology, Chinese Academy of Medical Sciences, Nanjing, China; ^4^Key Laboratory of Liver Transplantation, Chinese Academy of Medical Sciences, Nanjing, China; ^5^Key Laboratory of Living Donor Liver Transplantation, National Health Commission (NHC), Nanjing, China; ^6^Jiangsu Collaborative Innovation Center of Biomedical Functional Materials, College of Chemistry and Materials Science, Nanjing Normal University, Nanjing, China; ^7^Jiangsu Key Laboratory of Cancer Biomarkers, Prevention and Treatment, Collaborative Innovation Center for Personalized Cancer Medicine, Nanjing Medical University, Nanjing, China

**Keywords:** microRNA-98, hepatic stellate cell, liver fibrosis, HLF, HIF-1α

## Abstract

Liver fibrosis is a major endpoint of patients with chronic liver diseases. The molecular mechanisms behind liver fibrosis remain largely unknown. Many studies have indicated the role of microRNA (miRNA) in hepatic tumorigenesis. But the role of miRNA in liver fibrosis is little known. Activated hepatic stellate cells (HSCs) can secret extracellular matrix proteins (ECM) and are the major contributors to liver fibrosis/cirrhosis. Here, a microarray assay of quiescent and transforming growth factor β1 (TGF-β1) activated HSCs indicated that miR-98 might play a crucial role in liver fibrosis. We found that miR-98 was significantly downregulated in activated HSCs. miR-98 overexpression inhibited HSCs activation. Furthermore, we hypothesized that miR-98 regulated hepatic leukemia factor (HLF) expression by binding to the 3′ UTR of its mRNA directly, as evidenced by luciferase reporter assay. HLF overexpression increased HSCs activation by inducing hypoxia inducible factor-1 alpha (HIF-1α) expression, resulting in the activation of TGF-β/Smad2/3 signaling pathway. Besides, low expression of miR-98 was also found in liver tissues from various fibrotic murine models, including carbon tetrachloride (CCl_4_), bile duct ligation (BDL), and high-fat diet (HFD)-induced liver fibrosis. miR-98 overexpression *in vivo* by ago-miR-98 injection could attenuate CCl_4_-, BDL-, and HFD-induced murine hepatic fibrosis. Meanwhile, miR-98 overexpression suppressed HLF expression and reduced fibrosis marker expression. Collectively, our study demonstrates that miR-98 suppress HSCs activation by targeting HLF directly and interacting with HIF-1α/TGF-β/Smad2/3 signaling pathway, which may be an effective therapeutic target for liver fibrosis.

## Introduction

Liver fibrosis is a pathophysiological process and long-term persistent liver fibrosis can develop into cirrhosis, and hepatic carcinoma (Bataller and Brenner, 2005). Liver fibrosis is a wound-healing process characterized by a loss of liver architecture, attendant functional failure and the development of life-threatening complications (Friedman, 2008). Activated hepatic stellate cells (HSCs) produce mass of extracellular matrix (ECM), which could result in liver fibrosis (Higashi et al., 2017). Moreover, tissue inhibitor of metalloproteinases (TIMPs) are overexpressed, which inhibit the activation of ECM-removing matrix metalloproteinases (MMPs) (Gandhi, 2017). Multiple mediators, including transforming growth factor β (TGF-β) (Tang et al., 2017), reactive oxygen species (ROS) (Das et al., 2017), tumor necrosis factor α (TNF-α) (Osawa et al., 2013), and platelet-derived growth factor (PDGF) (Wilhelm et al., 2016), all induce HSC activation/proliferation. Regardless of above factors correlating with liver fibrosis intimately, the mechanism of hepatic stellate cell activation in liver fibrosis needs further exploration.

MicroRNAs (miRNAs) are characterized by single-stranded, small (22- to 25-nt), non-coding RNA molecules regulating cell differentiation, proliferation and survival by binding to complementary target mRNAs, leading to mRNA translational inhibition or degradation (Bartel, 2004; Rupaimoole and Slack, 2017). The effects of miRNAs on HSCs activation and transdifferentiation have been shown by many studies, which is crucial for liver fibrogenesis. miR-16 attenuates liver fibrosis and inhibit HSCs activation by suppressing autophagy (Kim et al., 2018). miR-455-3p alleviates hepatic stellate cell activation and liver fibrosis by suppressing HSF1 expression (Wei et al., 2019). miRNA-214 promotes hepatic stellate cell activation and liver fibrosis by suppressing Sufu expression (Ma et al., 2018). miRNA-125b promotes hepatic stellate cell activation and liver fibrosis by activating RhoA signaling (You et al., 2018). However, important miRNAs associated with liver fibrosis remain largely unrevealed.

In our study, we investigated the potential regulatory mechanism of candidate miR-98 in liver fibrogenesis. We found that miR-98 was significantly downregulated in activated HSCs and fibrotic liver tissues from patients, as evidenced by fibrotic murine models. miR-98 overexpression suppressed HSCs activation. Further experiments demonstrated that miR-98 regulated liver fibrosis by targeting HLF directly and suppressing its expression. miR-98 overexpression *in vivo* by ago-miR-98 injection could mitigate murine hepatic fibrosis. Collectively, our study demonstrates that miR-98 plays a pivotal role in liver fibrosis by targeting HLF signaling, which may be an effective therapeutic target.

## Materials and Methods

### Culture and Activation of Human HSC Line LX-2

Hepatic stellate cell line LX-2 were obtained from the Cell Center of Shanghai Institutes for Biological Sciences. Although, the study of stellate cell behavior has been gained through animal models and primary HSCs isolation, which undergo spontaneous activation that correlates closely with their response *in vivo*. Microarray analyses showed strong similarity in gene expression between primary HSCs and LX-2 (98.7%). LX-2 show strong viability in serum free media and high transfectability (Xu et al., 2005). DMEM containing 10% fetal bovine serum, 100 U/mL streptomycin sulfate, and 100 U/mL penicillin G sodium salt (Gibco, Carlsbad, CA, United States) were used to culture cells. LX-2 cells were subjected to TGF-β1 (10 ng/ml) for 0, 6, 12, and 24 h in serum-free DMEM for activation, respectively.

### Western Blot Assay

Proteins were extracted from liver tissues and cells with ice-cold lysis buffer (50 mM Tris, 0.1% sodium dodecyl sulfate, 150 mM Nacl, 1% Triton-100, 1% sodium deoxycholate). The following primary antibodies were used to incubate membranes: HLF (Abcam, Cambridge, MA, United States), HIF-1α, α-SMA, Collagen-I, TIMP-1, TGF-β, p-Smad2, Smad2, p-Smad3, Smad3, β-actin rabbit mAbs (Cell Signaling Technology, MA, United States). The reactions were detected with HRP-conjugated goat anti-rabbit immunoglobulin G (IgG) (Cell Signaling Technology, MA, United States) secondary antibodies.

### Quantitative RT-PCR

Total RNA was purified from liver tissues or cells using TRIzol reagent (Invitrogen, Carlsbad, CA, United States). The Transcriptor First Strand cDNA Synthesis Kit (Roche, Indianapolis, IN, United States) was used to perform reverse transcription. TaqMan miRNA assay system (Life Technologies Corporation, Shanghai, China) was used to detected the levels of U6 and miR-98. Gene expression was measured by qRT-PCR using SYBR green (Life Technologies, Grand Island, NY, United States). Results were normalized to β-actin expression and miR-98 to U6 snRNA, respectively. The primers used in this study were shown in Supplementary Table S1.

### miR-98 and Virus Transfection

The scrambled miRNA served as negative control (miR-SCR). miR-98-mimics were purchased (Gema, Shanghai, China). HLF knockdown and control lentiviruses (designated as Sh-HLF and Control) were purchased (Cyagen, Guangzhou, China). The RNAi target sequence was as follows: GCTGGGCAAATGCAAGAACAT. Lentivirus were transfected into LX-2 cells with Lipofectamine 2000 (Invitrogen, Carlsbad, CA, United States). Adenoviruses (ViaGen, Shandong, China) expressing FLAG-HLF, FLAG, HIF-1α and Vector (designated as Ad-HLF, Ad-Con, Ad-HIF-1α, and Ad-Con) were used. The anti-Flag antibody was used to recognize exogenous Flag-tagged HLF protein in Ad-HLF infected cells. Agomir control (CON) and ago-miR-98 (Gema, Shanghai, China) were injected into mice at 20 nmol/200 μl via tail injection. Mice were injected with agomir control and ago-miR-98 twice a week from 2 weeks after CCl4 injections or HFD. Mice were sacrificed at the end of the treatment. For BDL, mice were injected with ago-miR-98 and agomir control twice a week via tail vein after BDL. Two weeks later, mice were sacrificed and liver samples were collected for further experiments.

### Microarray Analyses

Total RNAs were extracted from LX-2 cells (treated with TGF-β1 for 0 and 24 h) with TRIzol reagent (Life Technologies, Grand Island, NY, United States). MirVana miRNA Isolation Kit (Life Technologies, Grand Island, NY, United States) was used to purify RNAs. Microarray experiments and analysis were performed by Shanghai Biotechnology Corporation (Shanghai, China). Agilent Feature Extraction software (version 10.7) was used to analyzed the scanned images.

### Flow Cytometric (FCM)

Cell Cycle Analysis Kit (Beyotime, Shanghai, China) was used to perform cell cycle experiments according to the manufacturer’s instructions. Briefly, trypsin was used to digest LX-2 cells. Then, LX-2 cells were centrifuged at 1,000 rpm for 5 min. 70% ethanol was used to fix cells prior to storage at −20°C overnight. Before FCM detection, RNase (50 μg/ml) was used to incubate cells. Cell cycle analysis was performed with PI staining solution (500 μl) to stain cells for 15 min at room temperature. PI (Sigma, Saint Louis, MO, United States) and Annexin V-FITC (BD Biosciences, San Diego, CA, United States) was used for apoptotic cells staining according to the manufacturer’s instructions.

### Murine Fibrosis Models

Eight-weeks-old male C57BL/6J mice were housed during the specific pathogen-free conditions with access to properly sterilized water and food. Liver fibrosis models were established through BDL (for 2 weeks) or CCl4 (10% in olive oil, 2 ml/kg, twice a week for 8 weeks). HFD (carbohydrates, 20.3%; fat, 61.6%; protein, 18.1%; D12492, Research Diets, New Brunswick, NJ, United States) and negative control (NC) diet (carbohydrates, 71.5%; fat, 10.2%; protein, 18.3%; D12450B, Research Diets) was used to induce hepatic steatosis model constantly for 24 weeks.

### Human Liver Samples

The fibrotic liver tissues were obtained from patients with hepatic fibrosis that underwent liver resection in the First Affiliated Hospital of Nanjing Medical University. The control liver tissues were the distal para-hemangioma normal tissues from patients undergoing surgical resection for hepatic hemangioma. The patient demographics and number of human samples analyzed were shown in Table 1.

**TABLE 1:** Demographic information of the study population.

	Number	Age (years)	Sex (male/female)	METAVIR score
Normal	25	46.56 ± 4.9	16/9	0
Fibrosis	33	48.73 ± 4.5	19/14	≥1

### Histology Analysis

Liver tissues were fixed in 4% paraformaldehyde. Liver tissue sections (4 μm thickness) were subjected to the Masson and Sirius Red staining for the extent of collagen deposition. For HFD-induced liver fibrosis, liver tissue sections were stained with Oil red O for lipid accumulation.

### Cell Proliferation and Migration Assays

For cell migration analysis, LX-2 cells (2 × 10^5^) were seeded into the upper chamber of Transwell with serum-free DMEM to detect cell migration. The lower chamber was added with DMEM containing 10% FBS which served as chemoattractant. The chamber was fixed after 2 h incubation. The mean number of cells per field of view were used to show cell counts. For cell proliferation analysis, 96-well plates were seeded with LX-2 cells (3 × 10^3^ cells per well). Cell Counting Kit-8 (Dojindo, Kumamoto, Japan) was used to detect ATP activity at specific time points.

### Immunohistochemical (IHC) and Immunofluorescence Staining

Tissue samples were fixed with 4% formalin and embedded in paraffin. Briefly, liver tissue sections were incubated with primary antibody of α-SMA (Cell Signaling Technology, MA, United States) or HLF (The International Cooperation Laboratory on Signal Transduction, EHBH, SMMU, China). HRP-Polymer-conjugated antibody was used as the secondary antibody. Subsequently, 3,3′-diaminobenzidine tetrachloride was used. The nuclei were counterstained with hematoxylin. HLF and α-SMA in LX-2 cells were identified by immunofluorescence using anti-mouse α-SMA mAb (Cell Signaling Technology, MA, United States) and anti-rabbit HLF pAb (The International Cooperation Laboratory on Signal Transduction, EHBH, SMMU, China), followed by incubation with secondary goat anti-mouse Texas Green-conjugated IgG or secondary goat anti-rabbit Texas Red-conjugated IgG (Sigma, St. Louis, MO, United States). The nuclei were further stained with DAPI. The slides were washed twice with PBS and examined with confocal microscopy (ZEISS, Oberkochen, Germany) according to the manufacturer’s instructions. Positive cells were blindly observed in 10 HPF/section (×400).

### Luciferase Reporter Assay

LX-2 cells were co-transfected with 0.12 μg wild-type or mutant reporter plasmid (Ambion, Austin, TX, United States) together with 40 nM of miR-98 mimics or scrambled miRNA (negative control) with Lipofectamine 3000 (Invitrogen, Carlsbad, CA, United States). LX-2 cells were also transfected with Renilla luciferase expression plasmid (0.01 μg) as a reference control. LX-2 cells were collected and lysed at 48 h after transfection. The luciferase reporter assay was conducted using the Dual-luciferase Reported Assay System (Promega, Madison, WI, United States) according to the manufacturer’s instruction.

### Chromatin Immunoprecipitation Assays

Magna ChIP HiSens Kit (Millipore, Bedford, MA, United States) was used to perform the chromatin immunoprecipitation (ChIP) assay according to the manufacturer’s instructions. The chromatin was immunoprecipitated with IgG and anti-Flag-HLF antibodies. The DNA was purified, and subjected to PCR to analyze the bound sequences. The primers used were as follows: HIF-1α, forward: 5′ TTAGTAGACAAGGTGAGTTCC 3′, reverse: 5′ CGTTGCTCAGATGTGTTAC 3′.

### Statistical Analysis

Data were expressed as the mean ± SEM. One-way ANOVA or Student’s *t* test was used to assess statistical significance. All analysis were performed with Stata software (version 11.0). *P* < 0.05 (two-tailed) was considered statistically significant.

## Results

### miR-98 Is Downregulated in Activated HSCs

**FIGURE 1:**
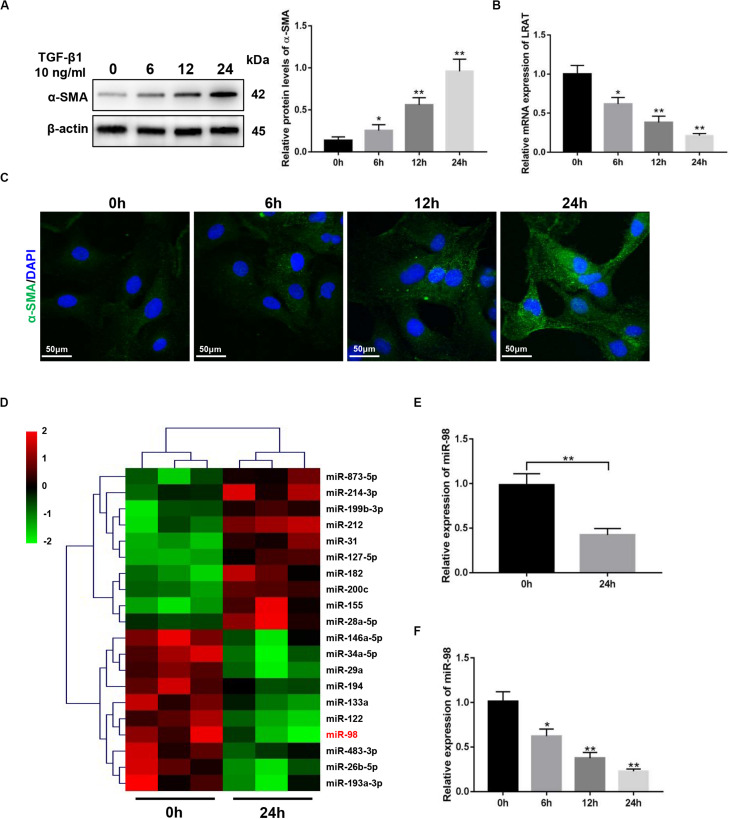
miR-98 ismademade downregulated in activated HSCs. **(A)** The protein level of α-SMA was upregulated in activated LX-2 cells treated with 10 ng/mL TGF-β1 in a time-dependent manner. Representative of three experiments. **(B)** The mRNA level of LRAT was downregulated in activated LX-2 cells treated with 10 ng/mL TGF-β1 in a time-dependent manner. Representative of three experiments. **(C)** Immunofluorescence staining for α-SMA (green) showed a increase in LX-2 cells treated with 10 ng/mL TGF-β1 in a time-dependent manner. Representative of three experiments. **(D)** Microarray analysis for miRNA expression was performed using total RNAs extracted from resting and activated LX-2 cells. **(E)** The expression level of miR-98 in LX-2 cells was examined by quantitative real-time PCR. **(F)** The expression level of miR-98 in activated LX-2 cells was examined in a time-dependent manner. *P < 0.05, **P < 0.01.

The expression level of a-smooth muscle actin (α-SMA) in activated HSCs (aHSCs) induced by TGF-β1 was detected first and showed a time-dependent increase in LX-2 cells (Figures 1A,C). The expression level of lecithin:retinol acyltransferase (LRAT) in activated HSCs (aHSCs) induced by TGF-β1, which is the physiological retinol esterification enzyme of the liver and is a potential and relevant tissue marker for quiescent HSC (Nagatsuma et al., 2009), was detected and showed a time-dependent decrease in LX-2 cells (Figure 1B). To examine the changes of miRNA expression profiles in activated HSCs, we performed miRNA microarray analysis on total RNAs extracted from LX-2 treated with 10 ng/mL TGF-β1 for 0 and 24 h. We found that 20 miRNAs were significantly differently expressed in activated LX-2 (Figure 1D). As shown in Figure 1D, miR-98 was one of the most significantly downregulated miRNAs. Reduced expression of miR-98 was validated by quantitative real-time PCR analysis (Figure 1E), which showed a time-dependent decrease in response to TGF-β1 in LX-2 cells (Figure 1F). These findings suggested that the expression of miR-98 was downregulated in activated HSCs.

### miR-98 Overexpression Suppresses the Activation and Proliferation of HSCs

**FIGURE 2:**
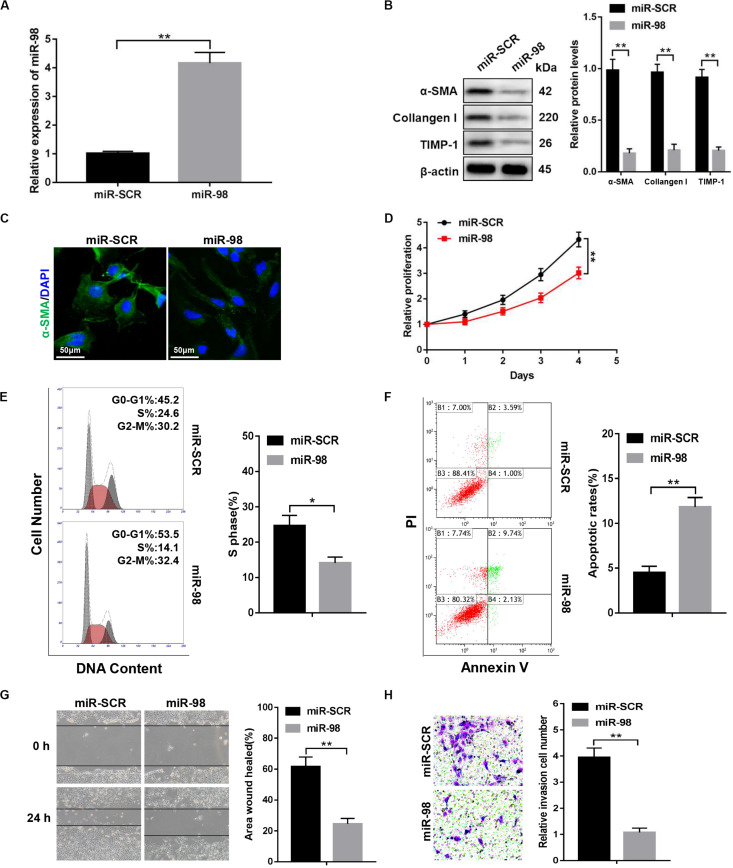
miR-98 overexpression suppresses the activation and proliferation of HSCs. **(A)** The expression level of miR-98 was examined in LX-2 cells after transfection with miR-98 mimics. **(B)** The protein levels of α-SMA, Collagen-I, and TIMP-1 were examined by western blotting. Representative of three experiments. **(C)** Immunofluorescence staining for α-SMA (green) was analyzed by confocal laser microscopy in LX-2 cells after transfection with miR-98 mimics. DAPI-stained nuclei, blue. Original magnification × 400; scale bars, 50 μm. **(D)** Proliferation of LX-2 cells transfected with miR-SCR and miR-98 was detected by CCK8 assay. **(E)** The cell-cycle distribution of miR-98-overexpressed LX-2 cells was detected by flow cytometry and the quantification. Representative of three experiments. **(F)** The cell apoptosis of LX-2 cells was detected by flow cytometry and the quantification. Representative of three experiments. **(G)** The migration capability of LX-2 cells transfected with miR-98 mimics or miR-SCR was measured using the wound-healing assay. Representative results from three independent experiments were shown. **(H)** The migration of the LX-2 cells transfected with miR-98 mimics or miR-SCR was compared using the Transwell assay, representative of three experiments. The number of cells was counted from different fields. Graph represents mean ± SEM. *P < 0.05, **P < 0.01.

To investigate whether ectopic expression of miR-98 in the HSC influenced HSC activation, we transfected LX-2 cells with miR-98 mimics (miR-98) or scrambled miRNAs (miR-SCR). The miR-98 levels were significantly higher in LX-2 cells transfected with miR-98 mimics (Figure 2A). The overexpression of miR-98 in LX-2 cells decreased protein levels of profibrotic markers, including α-SMA, Collagen-I, and TIMP-1 (Figure 2B). Accordingly, immunofluorescence analysis indicated a reduction of α-SMA in LX-2 cells treated with miR-98 mimics (Figure 2C). In addition, overexpression of miR-98 also significantly inhibited the cell proliferation and decreased the proportion of S phase cells (Figures 2D,E). Moreover, overexpression of miR-98 also led to increased apoptosis in LX-2 cells (Figure 2F). Furthermore, forced miR-98 expression significantly inhibited the migration capability of LX-2 cells (Figures 2G,H). Our results revealed that overexpression of miR-98 could inhibit the activation and proliferation of HSCs.

### miR-98 Targets HLF and Regulates Its Expression

**FIGURE 3:**
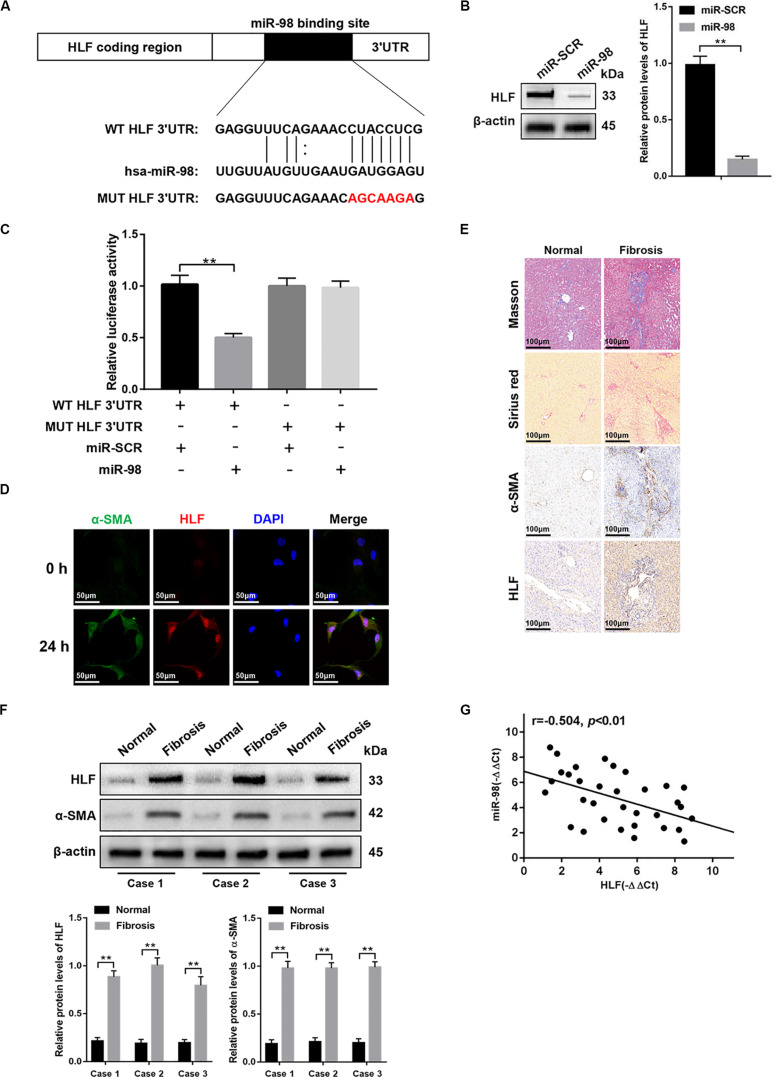
miR-98 target HLF and regulates its expression. **(A)** schematic drawing indicated the putative binding sites of miR-98 with respect to HLF. **(B)** The protein levels of HLF were detected by western blotting. **(C)** Dual-luciferase reporter assay of LX-2 cells transfected with WT HLF 3′ UTR or MUT HLF 3′ UTR reporter. **(D)** Dual immunofluorescence staining of LX-2 cells transfected with miR-98 mimics or miR-SCR using anti-α-SMA and anti-HLF antibodies. Representative of three experiments. The nuclei were counterstained with DAPI, original magnification × 400; scale bars, 50 μm. **(E)** Tissue sections of normal or fibrotic liver from patients were subjected to Masson staining, Sirius red staining and Immunohistochemistry (*n* = 6/group; original magnification ×200; scale bar = 100 μm). **(F)** The protein levels of HLF and α-SMA in normal or fibrotic liver tissues from patients were detected by western blotting. Representative of three experiments. **(G)** The correlation between HLF levels and miR-98 expression in patient fibrotic liver tissues was assessed using Pearson’s correlation analysis, *n* = 33. Graph represents mean ± SEM.

To further explore how miR-98 regulates HSC activation, we used bioinformatics software including miRanda, miRbase, and TargetScan. We found that the 3′-UTR of HLF contains putative binding sites for miR-98 (Figure 3A). A previous study has shown that inactivation of HLF could inhibit HSC activation and alleviated liver fibrosis (Xiang et al., 2018). Nevertheless, whether miR-98 could regulate HLF expression in HSCs and fibrotic liver has not yet been explored. Next, miR-98 mimics were transfected into LX-2 cells. As shown in Figure 3B, miR-98 overexpression inhibited HLF expression in LX-2 cells. Luciferase reporter gene assay further suggested that miR-98 mimics suppressed the luciferase activity of HLF with the wild-type 3′-UTR (WT HLF 3′-UTR), but not with its mutant 3′-UTR (MUT HLF 3′-UTR) (Figure 3C). To determine the role of HLF in liver fibrosis, we detected the expression of α-SMA and HLF in LX-2 cells activated by TGF-β1. Strikingly, dual immunofluorescence staining of LX-2 cells activated by TGF-β1 displayed a close co-localization of HLF and aHSCs marker α-SMA (Figure 3D). In addition, the expression levels of HLF were higher in liver tissues of patients with liver fibrosis than those in normal controls (Figures 3E,F). Moreover, the negative correlation between miR-98 levels and HLF expression was observed in patient fibrotic tissues (Figure 3G). These results suggested that miR-98 might target HLF in LX-2 cells and regulate liver fibrosis.

### miR-98 Regulates HSCs Activation Depending on HLF Expression

**FIGURE 4:**
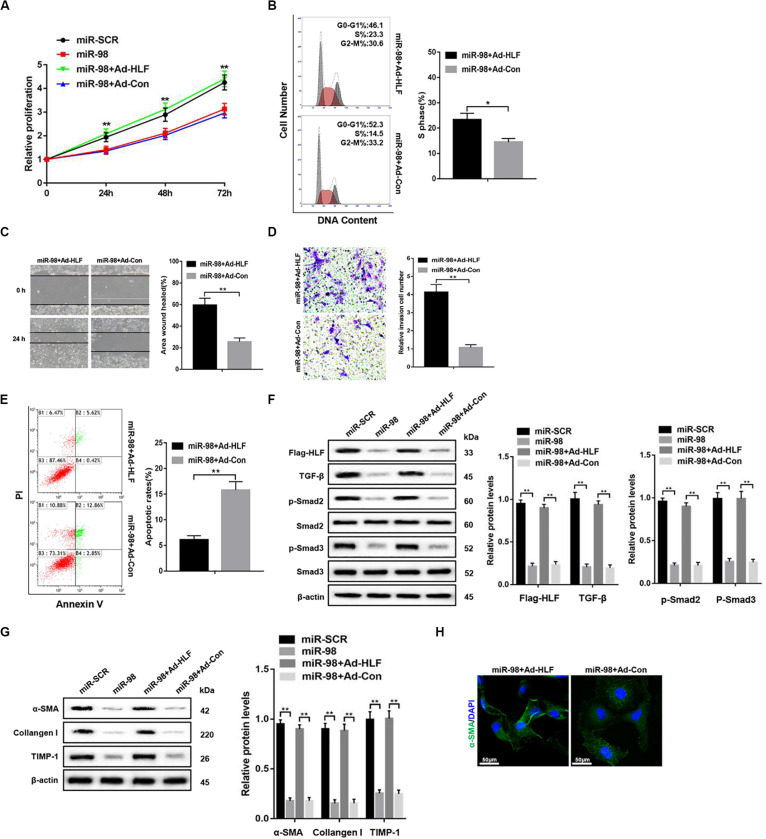
miR-98 regulates HSCs activation depending on HLF expression. **(A)** Proliferation of LX-2-pre-miR-98 cells transfected with Ad-HLF or Ad-Con was detected by CCK8 assay. **(B)** The cell-cycle distribution of LX-2-pre-miR-98 cells transfected with Ad-HLF or Ad-Con was detected by flow cytometry and the quantification. Representative of three experiments. **(C)** The migration capability of LX-2-pre-miR-98 cells transfected with Ad-HLF or Ad-Con was measured using the wound-healing assay. Representative of three experiments. **(D)** The migration of the LX-2-pre-miR-98 cells transfected with Ad-HLF or Ad-Con was compared using the Transwell assay, representative of three experiments. The number of cells was counted from different fields. **(E)** The cell apoptosis of LX-2-pre-miR-98 cells transfected with Ad-HLF or Ad-Con was detected by flow cytometry and the quantification. Representative of three experiments. **(F)** The expression levels of Flag-HLF, HIF-1α, TGF-β, p-Smad2, and p-Smad3 were examined in LX-2-pre-miR-98 cells transfected with Ad-HLF or Ad-Con. Representative of three experiments. **(G)** The protein levels of α-SMA, Collagen-I, and TIMP-1 were examined by western blotting in LX-2-pre-miR-98 cells transfected with Ad-HLF or Ad-Con. Representative of three experiments. **(H)** Immunofluorescence staining for α-SMA (green) was analyzed by confocal laser microscopy in LX-2-pre-miR-98 cells transfected with Ad-HLF or Ad-Con. Representative of three experiments. Graph represents mean ± SEM. *P < 0.05, **P < 0.01.

To further elucidate that miR-98 regulated HSC activation and proliferation by targeting HLF, we delivered Flag-tagged HLF into the cultured LX-2 cells using adenovirus to overexpress HLF in LX-2 cells transfected with miR-98 mimics. HLF overexpression could promote the cell proliferation and increase the proportion of S phase cells (Figures 4A,B). HLF overexpression significantly promoted the migration capability of LX-2 cells (Figures 4C,D) and led to decreased apoptosis in LX-2 cells (Figure 4E). Moreover, HLF overexpression upregulated the expression levels of Flag-HLF, TGF-β, and enhanced Smad2/3 activation in LX-2 transfected with miR-98 mimics (Figure 4F). In addition, HLF overexpression increased the expression of profibrotic markers including α-SMA, Collagen-I, and TIMP-1 (Figure 4G), as evidenced by increased α-SMA immunofluorescence staining (Figure 4H). These results indicated that the miR-98/HLF axis was involved in regulating HSC activation critically.

### miR-98 Regulates HSCs Activation Depending on HLF/HIF-1α Signaling Pathway

**FIGURE 5:**
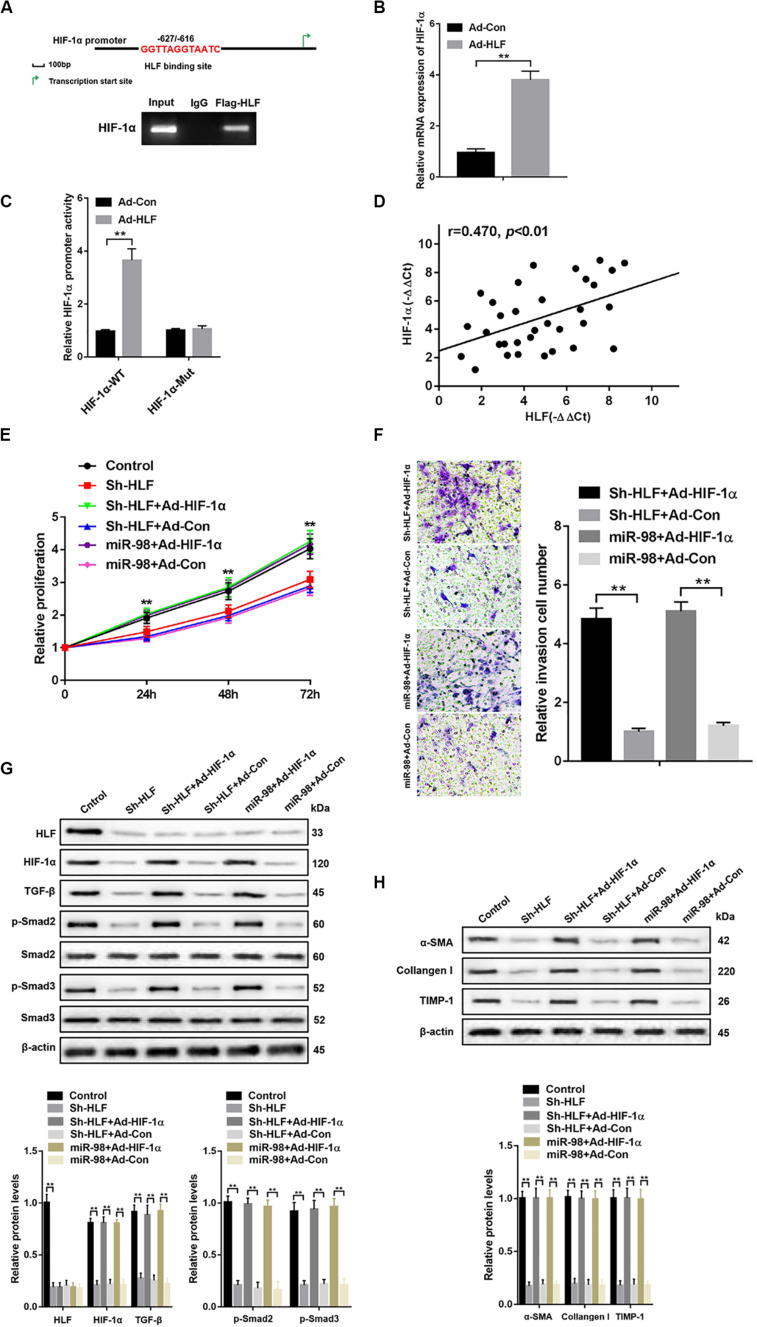
miR-98 regulates HSCs activation depending on HLF/HIF-1α signaling pathway. **(A)** Schematic diagram of the putative HLF binding site within the human HIF-1α promoter. The LX-2 cells infected with Ad-HLF were subjected to ChIP assay with anti-Flag or IgG antibody. Representative results from three independent experiments are shown. **(B)** HIF-1α mRNA expression in activated LX-2 cells transfected with Ad-HLF or Ad-Con. **(C)** The luciferase reporter activity of HIF-1α promoter (HIF-1α-WT) or the mutant of HIF-1α promoter (HIF-1α-Mut) was measured in activated LX-2 cells infected with Ad-HLF or Ad-Con. **(D)** The correlation between HLF levels and HIF-1α expression in patient fibrotic liver tissues was assessed using Pearson’s correlation analysis, *n* = 33. **(E)** Proliferation of LX-2-pre-Sh-HLF or LX-2-pre-miR-98 cells transfected with Ad-HIF-1α or Ad-Con was detected by CCK8 assay. **(F)** The migration of the LX-2-pre-Sh-HLF or LX-2-pre-miR-98 cells transfected with Ad-HIF-1α or Ad-Con was compared using the Transwell assay, representative of three experiments. The number of cells was counted from different fields. **(G)** The expression levels of HLF, HIF-1α, TGF-β, p-Smad2, and p-Smad3 were examined in LX-2-pre-Sh-HLF or LX-2-pre-miR-98 cells transfected with Ad-HIF-1α or Ad-Con. Representative of three experiments. **(H)** The protein levels of α-SMA, Collagen-I, and TIMP-1 were examined by western blotting in LX-2-pre-Sh-HLF or LX-2-pre-miR-98 cells transfected with Ad-HIF-1α or Ad-Con. Representative of three experiments. Graph represents mean ± SEM.

To further explore how HLF regulated HSC activation and proliferation, a putative homologous HLF binding site within the human HIF-1α promoter (−627/−616) was uncovered by bioinformatics analysis and verified by ChIP assays (Figure 5A). Consistently, overexpression of HLF increased HIF-1α expression in HSCs (Figure 5B). Next, the mutation of HLF binding site within HIF-1α promoter region abrogated the enhancement of HIF-1α promoter activity triggered by ectopic HLF expression (Figure 5C). Moreover, the correlation between HIF-1α levels and HLF expression was observed in patient fibrotic tissues (Figure 5D). To explore the role of HIF-1α in HSC activation, we used HIF-1α adenovirus to overexpress HIF-1α in LX-2 cells transfected with Sh-HLF or miR-98 mimics. HIF-1α overexpression significantly promoted the proliferation and the migration capability of LX-2 cells (Figures 5E,F). Furthermore, HIF-1α overexpression increased the levels of TGF-β and induced Smad2/3 activation compared with control (Figure 5G). Consistently, HIF-1α overexpression could increase the expression level of profibrotic marker α-SMA, Collagen-I, and TIMP-1 in LX-2 cells transfected with Sh-HLF or miR-98 mimics (Figure 5H). Our results indicated miR-98 regulates HSCs activation depending on HLF/HIF-1α signaling pathway.

### miR-98 Is Downregulated and HLF Is Overexpressed in Different Liver Fibrotic Models

**FIGURE 6:**
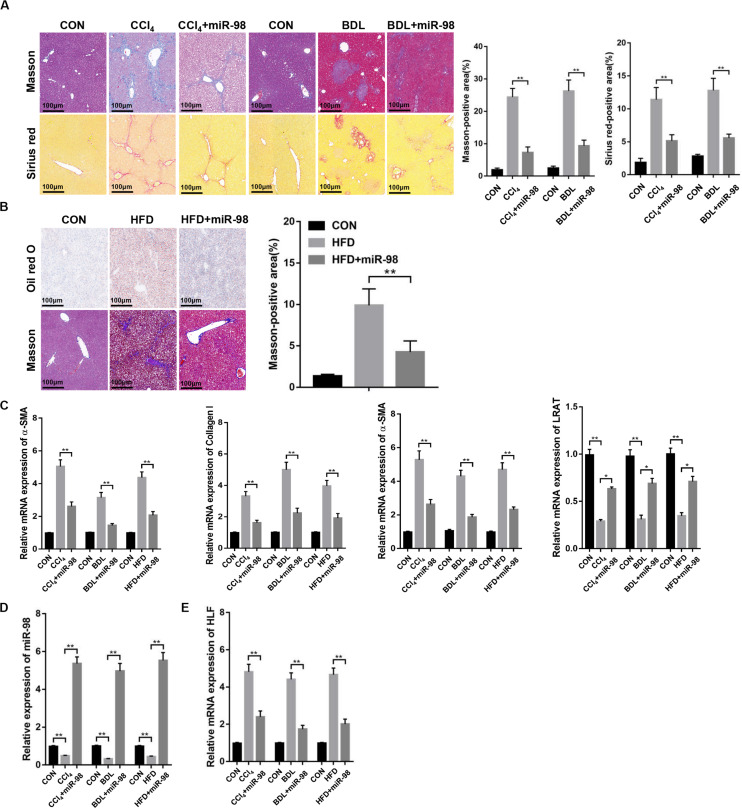
miR-98 is downregulated and HLF is overexpressed in different hepatic fibrosis models. **(A)** The sections of liver tissues from mice exposed to CCl_4_ for 8 weeks and BDL for 2 weeks or injected with agomir ago-miR-98 were subjected to Masson staining and Sirius red staining (*n* = 6 mice for each group; original magnification × 200; scale bar = 100 μm). **(B)** The sections of liver tissues from mice with HFD for 24 weeks or injected with agomir ago-miR-98 were subjected to Oil red O staining and Masson staining (*n* = 6 mice for each group; original magnification × 200; scale bar = 100 μm). **(C)** The expression levels of α-SMA, Collagen-I, TIMP-1 and LRAT were examined in liver tissues from the mice with CCl_4_-, BDL-, and HFD-induced liver fibrosis or injected with agomir ago-miR-98 by quantitative real-time PCR. *n* = 6 mice for each group. **(D)** The expression level of miR-98 was examined in liver tissues from mice with CCl_4_-, BDL-, and HFD-induced liver fibrosis or injected with agomir ago-miR-98 by quantitative real-time PCR. *n* = 6 mice for each group. **(E)** The expression level of HLF was examined in liver tissues from mice with CCl_4_-, BDL-, and HFD-induced liver fibrosis or injected with agomir ago-miR-98 by quantitative real-time PCR. *n* = 6 mice for each group. Graph represents mean ± SEM. *P < 0.05, **P < 0.01.

Then, mice were treated with CCl_4_, BDL or HFD to develop different liver fibrotic models. Masson and Sirius red staining showed the exacerbated liver fibrosis and increased collagen deposition in mice treated with CCl_4_, BDL (Figure 6A). The results of Oil red O and Masson staining revealed the increased steatosis and liver fibrosis after HFD treatment (Figure 6B). The overexpression of fibrotic markers, including α-SMA, Collagen-I, and TIMP-1, were also detected in the fibrotic liver tissues from CCl_4_-, BDL-, and HFD-treated mice compared to those of control (Figure 6C). Quantitative real-time PCR suggested that the mRNA levels of miR-98 in different fibrotic liver tissues was significantly downregulated (Figure 6D). Furthermore, the high expression of HLF was also detected in the fibrotic liver tissues from CCl_4_-, BDL-, and HFD-induced murine liver fibrosis (Figure 6E). To further study the role of miR-98 in hepatic fibrosis *in vivo*, the agomir control (CON) and ago-miR-98 (miR-98) were injected into CCl_4_-, BDL-, and HFD-treated or untreated mice. Masson and Sirius Red staining indicated that miR-98 overexpression attenuated hepatic fibrosis *in vivo* (Figures 6A,B). Quantitative real-time PCR suggested that miR-98 overexpression suppress the mRNA expression of α-SMA, Collagen-I, TIMP-1, and LRAT (Figure 6C). The transfection efficiency of miR-98 was detected by quantitative real-time PCR with liver tissues (Figure 6D). Quantitative real-time PCR showed significantly low HLF expression in the liver tissues from CCl_4_-, BDL-, and HFD-treated mice injected with ago-miR-98 (Figure 6E). These findings demonstrated that miR-98 was downregulated and HLF was overexpressed in different hepatic fibrosis models and played a pivotal role in the progression of liver fibrosis.

### miR-98 Alleviates Hepatic Fibrosis in Mice

**FIGURE 7:**
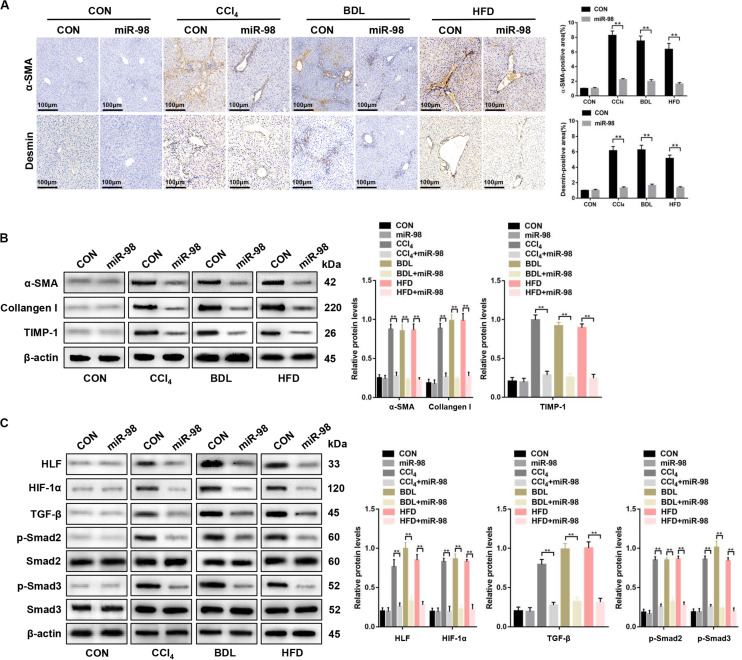
miR-98 suppresses HLF expression and alleviates hepatic fibrosis in mice. **(A)** The sections of liver tissues from mice injected with agomir control or ago-miR-98 with CCl_4_-, BDL-, and HFD-induced liver fibrosis were subjected to immunohistochemical staining (*n* = 6 mice for each group; original magnification × 200; scale bar = 100 μm). **(B)** The protein levels of α-SMA, Collagen-I, and TIMP-1 in liver tissues from untreated mice and mice treated with the agomir control and ago-miR-98 with CCl_4_-, BDL-, and HFD-induced liver fibrosis were examined by western blot. Representative of three experiments. **(C)** The protein levels of HLF, HIF-1α, TGF-β, p-Smad2, and p-Smad3 in liver tissues from mice treated with agomir control and ago-miR-98 with CCl_4_-, BDL-, and HFD-induced liver fibrosis were examined by western blot. Representative results from three independent experiments are shown. Graph represents mean ± SEM. **P < 0.01.

Next, α-SMA and desmin staining, the components of the HSC cytoskeleton, indicated that miR-98 overexpression attenuated hepatic fibrosis *in vivo* (Figure 7A). miR-98 overexpression reduced the expression levels of α-SMA, Collagen-I, and TIMP-1, HLF, HIF-1α, TGF-β, and inhibited Smad2/3 activation in liver tissues from CCl_4_-, BDL-, and HFD-treated mice after treatment with ago-miR-98 (Figures 7B,C). In conclusion, these findings demonstrated that miR-98 targeted HLF and attenuated hepatic fibrosis through the HIF-1α/TGF-β/Smad2/3 signaling pathway.

## Discussion

Liver fibrosis is a wound-healing response associated with chronic liver disease caused by infection, drugs, metabolic disorders, or immune attack, which engages a range of cell types and mediators to encapsulate injury (Friedman, 2008). Activation of HSCs transdifferentiating from quiescent cells into proliferative, fibrogenic myofibroblasts are recognized as the major contributors to liver fibrosis, which produce mass of ECM (Higashi et al., 2017; Tsuchida and Friedman, 2017).

The role of miRNAs in tumorigenesis has been studied extensively. Little is known is its role in fibrosis. HSCs can differentiate into myofibroblast-like cells upon fibrogenic injury of the liver and are the main cells contributing to liver fibrosis (Noetel et al., 2012). Studies have shown that miR-98 function differently in various types of tumors (Li et al., 2014; Liu et al., 2016; Zhang et al., 2017). Recently, a study demonstrated that miR-98 overexpression prevented rat pulmonary fibrosis (Gao et al., 2014). Furthermore, a study indicated that miRNA-98 inhibited TGF-β-induced differentiation and collagen production of cardiac fibroblasts (Cheng et al., 2017). Meanwhile, another study showed that miRNA-98 inhibited the cell proliferation of human hypertrophic scar fibroblasts (Bi et al., 2017). However, the mechanism that miR-98 regulates liver fibrosis remains unknown and needs further exploration.

In our study, microarray assay indicated that miR-98 was downregulated in activated HSCs. The overexpression of miR-98 effectively inhibited HSCs activation, proliferation and migration, as well as the expression of fibrogenic markers. Furthermore, miR-98 was downregulated in liver tissues from mice treated with CCl4, BDL, and HFD. miR-98 overexpression *in vivo* by ago-miR-98 injection could attenuate CCl_4_-, BDL-, and HFD-induced murine hepatic fibrosis. Our study indicated that miR-98 might inhibit liver fibrogenesis by regulating HSCs activation.

Next, we performed bioinformatics analysis with softwares including miRanda, miRbase, TargetScan and predicted that hepatic leukemia factor (HLF) might be the target bound by miR-98. Luciferase reporter assay further verified our hypothesis. HLF is a transcription factor of the proline and acidic amino acid-rich basic leucine zipper family. Previous study indicated that HLF regulated neurotransmitter metabolism in the brain (Mitsui et al., 2001; Gachon et al., 2004), xenobiotic detoxification in the liver (Gachon et al., 2006), and renal function (Zuber et al., 2009). A study demonstrated that HLF promoted hepatic stellate cell activation to aggravate liver fibrosis (Xiang et al., 2018). Our study indicated that the expression of HLF was high in liver tissues from patients with liver fibrosis. Recently, a study had shown that HLF increased HIF-1α gene expression and maintained cell proliferation, respiration and glycolysis (Otto and Fandrey, 2008). HIF-1α is a transcription factor functioning as a main regulator of oxygen homeostasis in all metazoan species, which controls oxygen metabolism (Semenza, 2014). Study demonstrated that HIF-1α enhanced synthesis of ECM components, leading eventually to the development of fibrosis (Sun et al., 2013). Another study suggested that HIF-1α might affect cellular redox status, which in turn regulated enzymes involved in collagen cross-linking and stabilization (Mariman and Wang, 2010). Therefore, we examined the expression of HIF-1α in liver fibrosis. We found that overexpression of HLF induced hypoxia inducible factor-1 alpha (HIF-1α) expression. A study indicated that HIF-1α activated the TGF-β/SMAD3 pathway, which might promote kidney injury and upregulate genes related to fibrosis (Kushida et al., 2016). Another study demonstrated that HIF-1α facilitated the transition of dermal fibroblasts to myofibroblasts through the activation of the TGF-β/Smad3 signaling pathway, which increased the expression of α-smooth muscle actin (α-SMA) and collagens I and III (Zhao et al., 2017). TGF-β plays a pivotal role in the development of hepatic fibrosis, which correlates with increased extracellular matrix deposition (Xu et al., 2016). SMAD proteins, as transcription factors, had been studied extensively as pivotal intracellular effectors of TGF-β (Meng et al., 2016). SMAD3 and SMAD4 are pro-fibrotic, whereas SMAD7 are anti-fibrotic (Ding et al., 2013; Lei et al., 2016; Wei et al., 2019). SMAD2 and SMAD3 are strongly activated in liver fibrosis (Yao et al., 2012). Our study found that miR-98 could suppress HLF expression in HSCs and attenuate liver fibrosis by inhibiting the HIF-1α/TGF-β/Smad2/3 axis.

In conclusion, our findings indicated that the expression of miR-98 decreased both in activated HSCs and different liver fibrotic models. miR-98 overexpression inhibited HSCs activation and the expression of profibrotic markers by targeting HLF via inhibiting the HIF-1α/TGF-β/Smad2/3 signaling pathway. Furthermore, miR-98 agomir alleviated hepatic fibrosis and inhibited HLF expression in mice. Our study disclosed the protective role miR-98 played in liver fibrosis by targeting HLF and regulating a novel HIF-1α/TGF-β/Smad2/3 signaling pathway.

## Data Availability Statement

The raw data supporting the conclusions of this article will be made available by the authors, without undue reservation.

## Ethics Statement

The studies involving human participants were reviewed and approved by the Ethics Committee of the First Affiliated Hospital of Nanjing Medical University. The patients/participants provided their written informed consent to participate in this study.

## Author Contributions

LLu to the study concept, research design, and finalized the manuscript. QW, SW, HZ, LLi, SZ, CS, YS, and JQ performed the experiments. QW and SW analyzed the data and wrote the first draft of manuscript. HZ, LLi, SZ, CS, YS, and JQ participated in the data analysis and critical discussion. All authors had final approval of the submitted and published versions.

## Conflict of Interest

The authors declare that the research was conducted in the absence of any commercial or financial relationships that could be construed as a potential conflict of interest.
