# Supplementary material for: MicroRNA-98 Inhibits Hepatic Stellate Cell Activation and Attenuates Liver Fibrosis by Regulating HLF Expression
Source: Front Cell Dev Biol. 2020 Jun 19;8:513. doi: 10.3389/fcell.2020.00513 (PMC7316892; doi:10.3389/fcell.2020.00513)
Supplement: Supplementary file 1 [file Table_1.docx]

**Supplementary table 1: Primer sequences for the amplification**.

| Gene | Forward Primer（5′ → 3′） | Reverse Primer（5′ → 3′） |
| --- | --- | --- |
| miR-98(Human) | ATCCAGTGCGTGTCGTG | TGCTTGAGGTAGTAAGTTG |
| U6(Human) | ATTGGAACGATACAGAGAAGATT | GGAACGCTTCACGAATTTG |
| LRAT(Human) | TGATGCCCGACATCCTGTTG | ATGTTAGCTCCGTAGGCGAAG |
| HLF(Human) | CCACCTTTATCCCGCCTCC | TTTACTAAATGCGTCTTCGTGGT |
| HIF-1α(Human) | GAACGTCGAAAAGAAAAGTCTCG | CCTTATCAAGATGCGAACTCACA |
| β-actin(Human) | CATGTACGTTGCTATCCAGGC | CTCCTTAATGTCACGCACGAT |
| miR-98(Mouse) | GCTGAGGTAGTAAGTTGTATTG | CAGTGCGTGTCGTGGAGT |
| U6(Mouse) | GCTTCGGCACATATACTAAAAT | CGCTTCACGAATTTGCGTGTCAT |
| HLF(Mouse) | CATCCCGTCTCCGAACTGTAT | GACTCGGTGTATTGCGGTTTG |
| α-SMA(Mouse) | GTCCCAGACATCAGGGAGTAA | TCGGATACTTCAGCGTCAGGA |
| Collagen-I(Mouse) | GCTCCTCTTAGGGGCCACT | CCACGTCTCACCATTGGGG |
| TIMP-1(Mouse) | GCAACTCGGACCTGGTCATAA | CGGCCCGTGATGAGAAACT |
| LRAT(Mouse) | CCGTCCCTATGAAATCAGCTC | ATGGGCGACACGGTTTTCC |
| β-actin(Mouse) | GGCTGTATTCCCCTCCATCG | CCAGTTGGTAACAATGCCATGT |
